# RAD tag sequencing as a source of SNP markers in *Cynara cardunculus *L

**DOI:** 10.1186/1471-2164-13-3

**Published:** 2012-01-03

**Authors:** Davide Scaglione, Alberto Acquadro, Ezio Portis, Matteo Tirone, Steven J Knapp, Sergio Lanteri

**Affiliations:** 1 Di.Va.P.R.A. Plant Genetics and Breeding, University of Torino, via L. da Vinci 44, 10095 Grugliasco (Torino), Italy; 2 Institute for Plant Breeding, Genetics, and Genomics, University of Georgia, 111 Riverbend Rd., 30602 Athens, Georgia USA

## Abstract

**Background:**

The globe artichoke (*Cynara cardunculus *L. var. *scolymus*) genome is relatively poorly explored, especially compared to those of the other major *Asteraceae *crops sunflower and lettuce. No SNP markers are in the public domain. We have combined the recently developed restriction-site associated DNA (RAD) approach with the Illumina DNA sequencing platform to effect the rapid and mass discovery of SNP markers for *C. cardunculus*.

**Results:**

RAD tags were sequenced from the genomic DNA of three *C. cardunculus *mapping population parents, generating 9.7 million reads, corresponding to ~1 Gbp of sequence. An assembly based on paired ends produced ~6.0 Mbp of genomic sequence, separated into ~19,000 contigs (mean length 312 bp), of which ~21% were fragments of putative coding sequence. The shared sequences allowed for the discovery of ~34,000 SNPs and nearly 800 indels, equivalent to a SNP frequency of 5.6 per 1,000 nt, and an indel frequency of 0.2 per 1,000 nt. A sample of heterozygous SNP loci was mapped by CAPS assays and this exercise provided validation of our mining criteria. The repetitive fraction of the genome had a high representation of retrotransposon sequence, followed by simple repeats, AT-low complexity regions and mobile DNA elements. The genomic k-mers distribution and CpG rate of *C. cardunculus*, compared with data derived from three whole genome-sequenced dicots species, provided a further evidence of the random representation of the *C. cardunculus *genome generated by RAD sampling.

**Conclusion:**

The RAD tag sequencing approach is a cost-effective and rapid method to develop SNP markers in a highly heterozygous species. Our approach permitted to generate a large and robust SNP datasets by the adoption of optimized filtering criteria.

## Background

*Cynara cardunculus *(*2n *= *2x *= 34, haploid genome size ~1.08 Gbp [1]) an allogamous, highly heterozygous Asteraceae species, includes three *taxa*: the globe artichoke (var. *scolymus*), the cultivated cardoon (var. *altilis*) and their common progenitor the wild cardoon (var. *sylvestris*) [2]. Globe artichoke contributes significantly to the Mediterranean agricultural economy, and is also cultivated in South America, North Africa, China and USA. Over the past 30 years, a body of evidence has grown that plant-based foods can be effective for the alleviation of several chronic diseases, and globe artichoke in particular has been shown to produce a number of nutraceutically and pharmaceutically active compounds. Extracts from both globe artichoke and cultivated cardoon have exhibited hepatoprotective, anticarcinogenic, antioxidative and antibacterial qualities, and even an inhibition of cholesterol biosynthesis and LDL oxidation [3-6]. Finally, there is increasing interest in developing the species as an energy and oilseed crop [7-10].

Since the first linkage map produced for globe artichoke [11], a number of other segregating populations have been exploited for genetic mapping, including one generated from a hybrid between a globe artichoke and a cultivated cardoon genotype [12] and, more recently, one obtained by crossing globe artichoke with wild cardoon [13]. The recent development of a set of gene-based microsatellites [14] has aided the construction of consensus genetic maps [13,15,16]. However, these maps remains insufficiently densely populated for trait mapping and marker assisted selection. Current high throughput sequencing technology, which produces DNA sequence at a rate several orders of magnitude faster than conventional methods, is effective as a platform for SNP (single nucleotide polymorphism) discovery. A particularly efficient protocol, termed "restriction-site associated DNA" (RAD) [17], in combination with the Illumina Genome Analyzer sequencing device [18], discovers SNPs by sequencing a large set of restriction fragments [19-21]. Here we report the generation of genomic RAD tags from the three *C. cardunculus *accessions used as the parents for two of our mapping populations. The RAD tags were used to derive SNP markers some of which were then validated by a Cleaved Amplified Polymorphic Sequence (CAPS) assay. The identified SNPs could be useful to produce denser *C. cardunculus *genetic maps via high-throughput genotyping technologies. The RAD sequence has also been informative for characterizing the repetitive DNA component of the *C. cardunculus *genome, in particular allowing some inferences to be made regarding the contribution of DNA methylation in inhibiting its expansion.

## Results and Discussion

### RAD tag sequencing and *de novo *contig assembly

**Figure 1:**
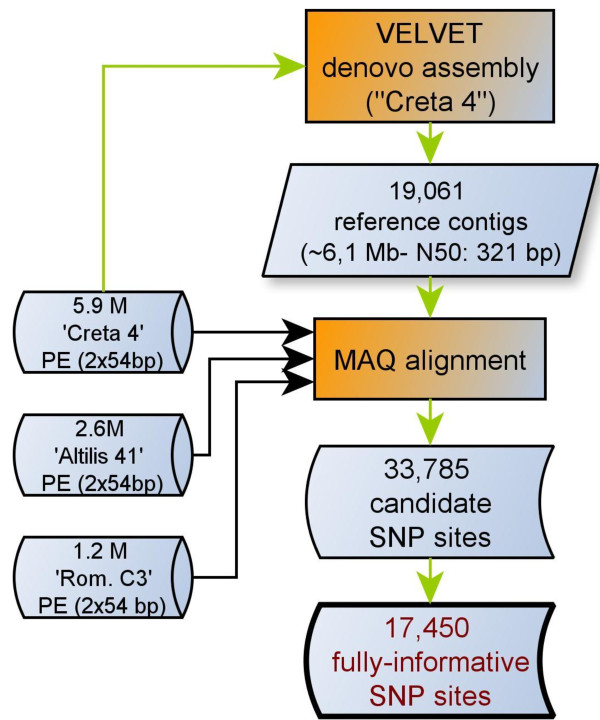
**Contig assembly and SNP discovery**. The *de novo *assembly was based on paired ends from "Creta 4". The alignment of paired ends was used to discover SNPs, using MAQ software. "Fully informative" SNP sites were those where sequence information was available for all three parental accessions.

The sequencing of the RAD libraries obtained from the three *C. cardunculus *accessions generated some 9.7 million reads (19.4 million paired ends), corresponding to ~1 Gbp of sequence. As reported previously [22], the distribution of reads was non-uniform across the three DNA samples, with 1.2 million reads achieved for globe artichoke, 2.6 million for cultivated cardoon and 5.9 million for wild cardoon. As a result, the wild cardoon variety was chosen as the basis for *de novo *contigs assembly. The sequence assembly pipeline (Figure 1) generated 19,061 reference contigs (Additional file 1), spanning 6.11 Mbp. The GC content of the sequence was about 37.4%, close to that prevailing in both *Arabidopsis thaliana *[23] and *Vitis vinifera *[24].

**Figure 2:**
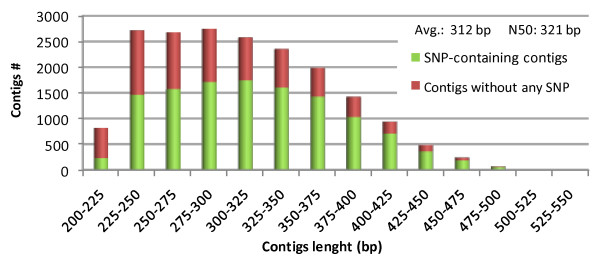
**Contig length distribution and the efficiency of SNP discovery**. Red bars represent the portion of contigs having no SNP identified, while green bars represent contigs harbouring at least one SNP.

As expected from the size-selection procedure used in the construction of the libraries, N50 was 321 bp and the mean contigs length was 312 bp (Figure 2). The reported contig length distribution is similar to the one described by Etter et al. [25], while other research (Baxter et al. [26], Willing et al. [27]) reported RAD contig lengths skewed towards the longer fragments. We hypothesize these differences to be related to coverage depth obtained during sequencing, as we used for our assembly ~6 M total reads, while Etter et al. used ~8 M reads, Baxter et al. ~13 M reads, and Willing et al. ~23 M reads. Furthermore, for the generation of RAD sites, we used a 6-cutter (PstI) enzyme while Etter et al. [25] and Baxter et al. [26] used SbfI, which is an 8-cutter. By targeting a reduced amount of genomic loci it's likely to gain a relative higher coverage which can promote the assembly of longer contigs. Alternative assemblies (i.e. more than one contig generated per RAD site, see "Materials and methods") accounted for less than the 7% of the RAD contig set, similarly to what reported by Willing et al. [27].

### Annotation and GO categorization of contigs

**Figure 3:**
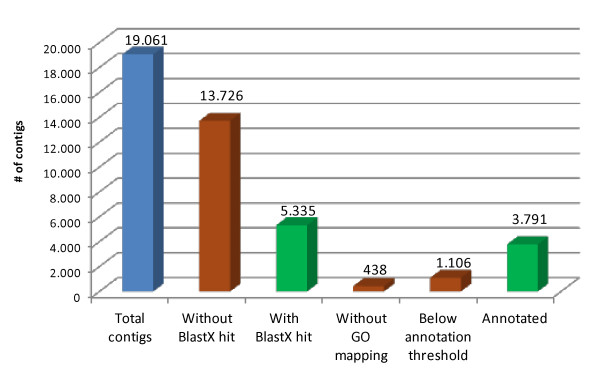
**Contig annotation**. Green bars represent sequences which either produced a BLASTX hit or passed the final annotation criteria. Brown bars represent contigs filtered out because of an absence of a BLASTX hit, no GO mapping or an annotation score below the threshold.

**Figure 4:**
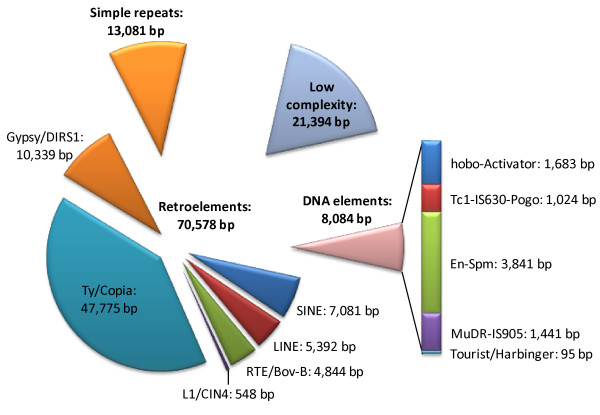
**Repetitive DNA in the RAD contigs**. The representation of known repetitive elements in *C. cardunculus *RAD sequence. Results generated by RepeatMasker analysis against the Repbase database.

The BLASTX search resulted in a top-hit list (composed by the first result of each BLAST output report) of protein sequences from *V. vinifera *(41% of the total hits), *Ricinus communis *(16%), *Populus trichocarpa *(15%) and *A. thaliana *(6%). Gene Ontology (GO) terms were assigned to 3,791 contigs (19.8%; Figure 3, Additional file 2). Most of the failed annotations (72.0%) applied to contigs lacking any BLASTX hit; of the remainder, 5.8% did not pass the annotation threshold and 2.3% resulted in no GO mapping. Overall, 5,335 contigs (28.0%) included at least one BLASTX hit with an E-value < 10e^-3^, with 3,554 of these (18.6%) recording an E-value < 10e^-15^. Despite the genome-wide RAD sampling, a noteworthy part of it may be likely represented by coding regions, since a methylation-sensitive enzyme (PstI) was used to produce the RAD-tag libraries [28]; notwithstanding the rather short length of the RAD contigs made it difficult to distinguish between sequences representing complete genes and pseudogenes. Enzyme codes were retrieved for 1,327 contigs, defining a unique set of 313 putative enzymatic activities, which were mapped onto KEGG reference pathways (Additional file 3). Within the repetitive DNA fraction (Figure 4), 1.2% of the sequences were derived from LTR retroelements, including Ty/Copia-like (0.8%) and Gypsy-like (0.2%). Transposable DNA element footprints accounted for a further 0.2% of the sequence. Note that this quantification of transposable element abundance could have been underestimated by the shortness of the RAD tag sequences which could affect search sensitivity.

### K-mer distribution analysis

**Figure 5:**
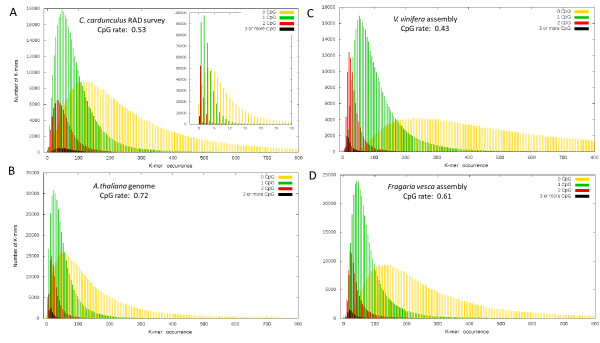
**Comparison of K-mer spectra in the *C. cardunculus *RAD contig assembly *vs *the full genomes of *A. thaliana*, *V. vinifera *and *F. vesca***. K-mer (k = 10) distribution for *C. cardunculus *(A) was evaluated both on pre-assembly sequence data (outer box) and contig sequences (inner box). K-mer populations have been split on the basis of their CpG content. × axis represents the number of occurrences of a given 10-mer; Y axis reports the amount of different 10-mers reporting that occurrence count.

With the aim to investigate whether the RAD sequencing was able to provide a representative and unbiased sample of the *C. cardunculus *genome, we compared the k-mers spectrum with other fully sequenced genomes. Moreover, we further investigated how CpG content correlate with the repetitive contents of the genome, as suggested by Chor et al. [29]. The frequency and distribution of 10-mers among the raw sequence and the assembled wild cardoon contigs were comparable to one another (Figure 5A). K-mers lacking CpG dinucleotides were over-represented in the more repetitive portion of the spectra (i.e. their distribution was right-skewed), while those bearing at least one CpG produced a more left-shifted distribution (Figure 5A). Results were confirmed by negative controls through the adoption of random dinucleotides, which did not show any preferential distributions of K-mers (Additional file 4). This outcome is consistent with the known correlation of CpG methylation with the repression of transposable elements [30,31]. A comparative study of other plant genomes showed that the *V. vinifera *genome has a higher frequency of zero-CpG K-mers (Figure 5C) than that of *A. thaliana *(Figure 5B), but that the *Fragaria vesca *K-mer distribution (Figure 5D) was rather similar to that obtained in *C. cardunculus *(Figure 5A). To futher investigate these trends, CpG rates [32] across the four dicot species were compared. While the CpG rate in the *C. cardunculus *RAD dataset was 0.53, 0.72 was calculated for *A. thaliana*, 0.43 for *V. vinifera *and 0.61 for *F. vesca *genomes [23,24,33]. Furthermore, the *A. thaliana *genome includes a 14% presence of repetitive elements [23], that in *V. vinifera *is 41% [24], and that in *F. vesca *22% [33]. Variations in CpG rates showed to be congruent with data derived from K-mer spectra analysis, since genomes harbouring higher rates of CpG reported less repetitive K-mer populations. This suggests a key contribution of DNA methylation in the inhibition of genome expansion due to repetitive element proliferation.

Altogether, our data suggest that the RAD procedure, despite its use of GC-rich recognition sites, has produced a random representation of the *C. cardunculus *genome, and shows that it represents a reliable means of assessing genome complexity.

#### SNP calling and classification

The paired ends generated for each mapping parent were aligned based on the reference contig set. This alignment detected 33,784 sequence variants, including 1,520 short indels, scattered over 12,068 contigs ('CcRAD1' dataset, Additional file 5). The overall SNP frequency was estimated to be 5.6 per 1,000 nucleotides, a level which is almost identical to that found in the non-coding regions of the *V. vinifera *genome (5.5 per 1,000 nucleotides) [34] and very similar to that uncovered among *Citrus *spp. ESTs (6.1 per 1,000 nucleotides) [35]. The estimation of SNP frequency using such high throughput sequencing data is, however, heavily dependent both on the number of genomes sampled, and on the extent (if any) of targeting and of genome coverage. The efficiency of SNP discovery was correlated with the length of the RAD tags (Figure 2). Contigs longer than 400 bp were associated with a 74% probability of finding at least one SNP, while this probability fell to 62% for contigs shorter than 400 bp. Setting as a criterion the need to identify SNPs informative for both mapping populations reduced the dataset size to 17,450 sequence polymorphisms distributed over 7,478 contigs ('CcRAD2' dataset, Additional file 6); of these, 16,727 were SNPs, and 723 were 1 or 2 nt indels. Some 57% of the contigs contained more than one polymorphic site, and non bi-allelic variants occurred at 959 sites. The number of heterozygous SNP loci was 1,235 in the globe artichoke parent, 2,868 in the cultivated cardoon and 5,069 in the wild cardoon. The loci were classified into those expected to segregate in a 1:1 ratio ("testcross markers"), and those in a 1:2:1 ratio ("intercross markers") (Table 1, Additional file 6). The lower number of reads generated from the globe artichoke template produced an under-representation of testcross markers, compared to the levels of informativeness observed previously for other marker types [36]. Moreover, genetic diversity across the three *taxa *might be responsible for *taxon*-specific RAD tags due to the absence of PstI restriction sites. In the final dataset ("fully informative" SNP sites, Additional file 6), the proportion of contigs including more than one informative marker was 26%.

**Table 1:** SNP mining results.

Filtering criteria	RAD-contigs count	SNPs count
Total SNPs mining (CcRAD1)	12,068	33,784
"Fully informative" RAD loci (CcRAD2)	7,478	17,450
Putative testcross markers (CcRAD2)	6,289	8,530
*"Romanesco C3" testcross over "Altilis 41"*	*724*	*883*
*"Altilis 41" testcross over "Romanesco C3"*	*1,541*	*2,210*
*"Romanesco C3" testcross over "Creta 4"*	*778*	*937*
*"Creta 4" testcross over "Romanesco C3"*	*3,246*	*4,500*
Common intercross markers (CcRAD2)	117	136

Two separated filtering criteria outcomes are reported (CcRAD1 and CcRAD2). Testcross and intercross markers evaluation was carried out exclusively on CcRAD2, representing SNP sites having sequence information for each of the three samples analyzed.

### CAPS markers conversion and linkage analysis

**Figure 6:**
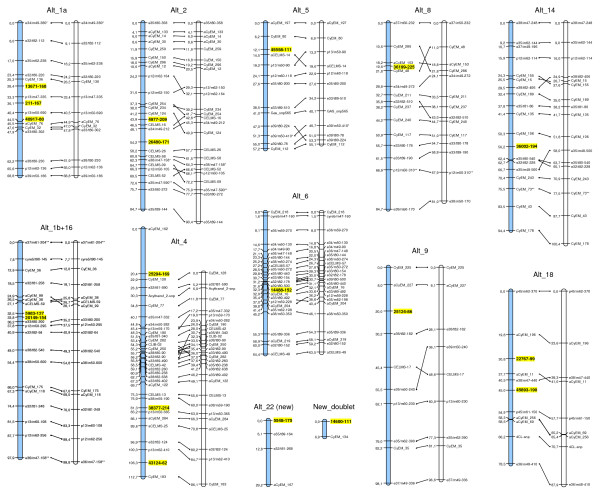
**Linkage analysis**. The linkage groups (LGs) forming the "Altilis 41" genetic map on which RAD-derived CAPs markers were positioned (yellow boxes). LG numbering is as given by Portis et al. [12]. LGs as they existed prior to the placement of CAPS loci are shown in white, while new LGs are blue.

A random selection of 24 SNPs was made from the CcRAD2 dataset in order to validate the SNP calls by conversion to a CAPS format. These assays were then used to genotype the globe artichoke × cultivated cardoon mapping population members [12]. Primer pairs were designed for testcross SNP loci expected to segregate only within cultivated cardoon (Table 2). Successful amplification was obtained for all the assays, and 19 out of the 24 segregated consistently with the predicted 1:1 ratio (Table 2). Three of the assays produced not readable patterns of segregation and were discarded, while other two showed no evidence of any restriction cleavage, suggesting either a false SNP call (e.g. assembly of paralogs, sequencing error) or failure in the assay (e.g. selective amplification of one allele). Among the 19 CAPS loci retained, none showed a significant level of segregation distortion (χ^2 ^≤ χ^2^_α = 0.1_); 17 loci were distributed over ten cultivated cardoon linkage groups, one (SNP site 5548-175) was associated to a previously linked pairs of markers and thereby generated a new LG (Alt_22), and CAPS 14600-111 was linked to the previously unmapped microsatellite locus CyEM-134 (Figure 6). CAPS loci 5983-127 and 20149-154 were most tightly linked with one another (1.3 cM on LG Alt_1b+16). The inclusion of these 17 loci generated only minor changes in locus order; some re-arrangements were induced in Alt_4 (CELMS-42, Δ10.0 cM), Alt_8 (CyEM_48, Δ10.8 cM and CyEM_286, Δ 11.2 cM) and Alt_9 (e39/m50-240, Δ19.4 cM). The mapping exercise confirmed that the RAD-derived SNP markers are suitable for genotyping purposes.

**Table 2:** CAPs markers conversion.

SNP-ID	Primer forward	Primer reverse	Reads ratio	Enzyme	Product size (bp)	Restriction site (bp)	"RomanescoC3" restriction produts	"Altilis 41" restriction products	Segregation pattern	Linkage groups
**211-167**	TCAACCCAATCTCGTCAGTG	CTTCATAGTGGCAGCCTGGT	10/30	*Eco*RV	372	162	372	372,210,162	Test cross	LG Alt_1a
**4977-209**	AAATCCCACATATGGAAATAGC	TCATGACACAAGGTGGAGACA	28/45	*Xmn*I	360	176	360	360,176,184	Test cross	LG Alt_2
**5548-175**	AATGCACAAACCAAGTGCAA	TGAGCTCATTCGGAGGAAAT	5/17	*Xmn*I	248	110	138,110	248,138,110	Test cross	New LG Alt_22
**5983-127**	TTGGTGGGTTTTAGACACCTTT	GTTAAACCCCCTGGATTGCT	3/5	*Taq*I	179	118	179	179,61,118	Test cross	LG Alt_1b
**13671-168**	TCTGGAGCATAAGAGGTAGGG	TTCAGTCGACTTCAAGGGAAC	13/20	*Fok*I	243	88	243	243,155,88	Test cross	LG Alt_1a
**14488-152**	AAAGCTTTTTCCCCTTTCC	AAGTGCGTATTTGATTGATTGA	22/51	*Mse*I	388	150	388	388,238,150	Test cross	LG Alt_6
**14600-111**	AAAAACACGCTCCTTCCATA	TGTCATCCCCATGAAAAAGC	7/12	*Bcc*I	290	97	290	290,193,97	Test cross	New doublet
**20149-154**	CCAGATGCAAATTGATACGTTG	GGATCTGCATTGAAACCTTGA	10/21	*Eco*RV	262	153	153,109	264,153,109	Test cross	LG Alt_1b
**22767-99**	CGGCACAACTAAGAGACAATCT	TTGGAGTATGTCTCGGGCTA	8/15	*Bcc*I	315	88	315	315,227,88	Test cross	LG Alt_18
**25124-86**	ACAAGGCCGGACCCTAAAC	TGGAACAGGAAGGACAGGTT	7/15	*Dra*I	288	71	288	288,217,71	Test cross	LG Alt_9
**25294-169**	GAGGAAACTTTTCCCCATCG	CCGTTGTTGTATGCCTCAAA	4/11	*Xba*I	327	159	212,159	327,212,159	Test cross	LG Alt_4
**25584-143**	ATTCGCCATGGAACAAGG	GCAGTCTAATGCTTCAACTGGT	12/29	*Taq*I	272	89	183,89	272,183,79	Unclear	-
**26480-171**	CGACAAACTCCCTCCATGTT	TGTGGTATTGATGGGGAACC	3/6	*Eco*RV	320	153	320	320,172,153	Test cross	LG Alt_2
**26420-81**	ACATCAACGCCAGCAAAGAT	TTCTTGTTTGAATCTCAAGTGC	5/18	*Xmn*I	281	76	205,76	281,205,76	Missing cut	-
**36002-194**	GCACAGGAAAATGTTGGTGTTA	GTCTTTGCAATTCCAATCAGA	5/16	*Dra*I	369	152	217,152	369,217,152	Test cross	LG Alt_14
**36199-225**	TGACCAGGTTTCAGGTATGTG	AACGTACAAATTCAAAGCACGA	7/11	*Bam*HI	398	221	221,177	398,221,177	Test cross	LG Alt_8
**38377-214**	AGAACCCGAAAACGTCTCCA	AGGACCTAATGCAGGTTCTGA	16/22	*Nde*I	451	203	451	451,248,203	Test cross	LG Alt_4
**38382-111**	CAGGGAGAATCCCTCTCTCA	CATATATTGGATGATCCCTTGG	4/9	*Dra*I	305	99	206,99	305,206,99	Unclear	-
**40917-80**	TGCTTCCCAATAGCCTCTAA	TGTGGTGATTTTGGACGTGT	7/13	*Fok*I	306	70	306	306,236,7	Test cross	LG Alt_1a
**43124-62**	TGATTATGCATCACCCCAAA	CACTTTTAATCCCAAAACAACC	9/19	*Taq*I	309	52	257,52	309,257,52	Test cross	LG Alt_4
**43867-147**	TGCATTTCTTCCTTGTGGTTC	ATGCTCCGTGAGGTTCGTAG	10/19	*Eco*RV	314	138	176,138	316,176,138	Unclear	-
**45558-111**	GGGAGAAGACCACGTAATTTGA	GTTTATTTCCGTCCCCAGGT	10/19	*Fok*I	294	122	172,122	294,172,122	Test cross	LG Alt_5
**45893-190**	TCATTGGTCTTGCAGTTGGA	ACTTGGGCTGTAGCTTGACG	8/13	*Taq*I	344	176	176,168	344,176,168	Test cross	LG Alt_18
**45900-239**	GGACAGTTTTGAGAAATGGTCT	TCACACGGTTTTGCAATCTC	2/6	*Eco*RV	306	203	306	306,203,103	Missing cut	-

CAPs markers conversion of 24 RAD loci randomly selected among CcRAD2. SNP-ID identifies the RAD contig name and the original SNP position, respectively separated by "-". Reads ratio refers to the number of occurrence of a nucleotide differing from the consensus sequence. Bands present only in the "Altilis 41" parental line are underlined. Linkage groups are reported according to the reference map of cultivated cardoon [12].

## Conclusion

In crop species where the number of markers available to date is limiting, the use of high throughput sequencing to generate large numbers of genetically informative assays can make a valuable and rapid contribution to linkage mapping, and its major downstream application, marker-assisted selection. RAD tag sequencing based on the Illumina platform has proven to be a highly reliable and cost-effective means of SNP discovery. We were able to identify thousands of putative SNP markers in this way, and the majority of a random sample of 24 was fully validated through conversion to CAPS assays and linkage analysis. Furthermore, the reduction in template complexity generated by the RAD approach greatly facilitates its implementation in mapping-by-sequencing approaches.

A large proportion of the methylation present in DNA occurs in the form of CpG dinucleotides, and there is little evidence for negative selection against these in the many genomes which have been analysed to date [32,37]. Acquiring genome-wide sequence has given a glimpse of the genome complexity present in *C. cardunculus*. Even though the RAD tags represent only a sample of the genome as a whole, it was clear that there exists a relationship between the frequency of CpG dinucleotides and the level of sequence repetitiveness, consistent with the known role played by methylation in controlling genome expansion due to transposable element activity [30,31].

## Methods

### Plant material and RAD tag sequencing

Genomic DNA was extracted from the leaf of the three *C. cardunculus *accessions, following the protocol described by Lanteri et al. [38]. The three accessions have been used as parents of two F_1 _populations, made by crossing globe artichoke variety "Romanesco C3" as female with either the cultivated cardoon variety "Altilis 41" or the wild cardoon accession "Creta 4" as male [11]. "Romanesco C3" is a late-maturing variety, which forms large purple-green capitula, each bearing violet coloured florets; "Altilis 41" was selected at the University of Catania [11] on the basis of its biomass yield potential; its foliage is grey and its florets white. "Creta 4" was collected from a wild population in Crete; it produces a large number of capitula, forms green-violet bracts and violet florets. Each DNA sample was processed into a separate RAD libraries as reported by Baird et al. [17]. Briefly, 300 ng DNA were digested with 20 U of PstI (New England Biolabs, NEB) for 60 min at 37°C in a 50 μl reaction, after which the reactions were heat inactivated by holding at 65°C for 20 min. A 2.5 μL aliquot of 100 nM P1 adaptor (a modified Illumina adapter) [18] was added to each sample along with 1 μL 10 mM ATP (Promega), 1 μL 10x NEB Buffer4, 1,000 U T4 DNA ligase (Enzymatics, Inc) and 5 μL H_2_O, and the reaction was incubated at room temperature for 20 min, ending with a heat inactivation step (65°C/20 min). The reactions were then pooled and sheared to an average length of 500 bp using a Bioruptor (Diagenode). The sheared DNA was separated by electrophoresis through a 1.5% agarose gel, and fragments in the 300-800 bp range were isolated using a MinElute Gel Extraction kit (Qiagen). The End-Repair mix (Enzymatics, Inc.) was used to blunten the dsDNA ends, and the samples were re-purified using a MinElute column (Qiagen), following which 15 U Exo-Klenow (Enzymatics, Inc.) were added and the sample incubated at 37°C to generate 3'-adenine overhangs. After subsequent purification, 1 μL 10 μM P2 adapter (a second modified Illumina adapter) [18] was ligated and the sample purified as above. The concentration of DNA in the eluate was quantified using a Qubit fluorimeter, and a 20 ng aliquot was used for a 100 μL PCR comprising 20 μL Phusion Master Mix (NEB), 5 μL 10 μM P2 and H_2_O. The 18 cycle PCR amplification regime followed the recommendation of the manufacturer (NEB). After this PCR, the samples were separated by electrophoresis once again through a 1.5% agarose gel, and fragments in the 300-700 bp range were excised from the gel and diluted to 3 ng/μL. The material was analysed on an Illumina Genome Analyzer IIx following the paired ends (2x 54 bp) genomic DNA sequencing protocol suggested by the manufacturer.

### RAD contig assembly

The sequences were sorted according to their multiplex identifier tag. A RAD LongRead^® ^contig assembly was generated by a set of algorithms developed at Floragenex Inc. Sequences having more than 5 bases with poor Illumina quality scores (Phred10 or lower) were discarded. Paired reads were collapsed into sequence "clusters" on the basis of single ends (SE) sharing 100% sequence identity. To maximize assembly efficiency, a minimum of 25x and maximum 400x sequence coverage at RAD SE reads were imposed. The variable paired end sequences for each common SE were extracted using the filtered sequence set and compiled for the LongRead^® ^contig construction, using a modified version of the Velvet sequence assembler (v. 1.0.04) [39] and testing several k-mers in graph construction for each RAD contig. After analysis of the first-pass assembly from each template, "Creta 4" was selected as the reference sequence set. Additional filters were then applied to remove short contigs (< 100 bp in length), low paired end coverage (< 4.0x) or ambiguous contigs (containing N's homopolymers). If more than a single contig (NODE1) was assembled for a given RAD locus, alternative ones were retained in the dataset and labelled accordingly (NODE2, NODE3).

### Contig annotation and categorization

RAD contigs were annotated using Blast2GO software [40], and were submitted to the NCBI nr protein database where an E-value of 10e^-3 ^or lower were retrieved (20 best hits recorded). Gene names and GIs (gene identifiers) were assigned according to NCBI guidelines, and PIR (Protein Information Resource) identifiers in reference to UniProt, SwissProt, TrEMBL, RefSeq, GenPept and PDB. The annotation was obtained by applying the formula embedded in Blast2GO [40], setting a threshold score of 55. In the Blast2Go pipeline, GO terms are "transferred" to query sequences only whether a score threshold is reached. This score is calculated basing on both sequence similarity and presence of children node in the directed acyclic graph (DAG). Therefore, in this scenario the first e-value cut-off is used only for the purpose of "collecting" GO-terms, while other more stringent criteria are ruling whether transfer these terms to our sequences. Enzyme codes were retrieved from GO tables and mapped onto KEGG pathways. Transposable elements were detected using RepeatMasker v3.2.9 software http://www.repeatmasker.org, based on the RMBlast algorithm. Default parameters (except for -s flag) were used to search against *Viridiplantae *repeats.

### K-mer distribution and CpG suppression

K-mer distribution and CpG suppression were analyzed using a Python script to split K-mer counts generated with Jellyfish [41]. The whole genome assemblies of *A. thaliana*, *V. vinifera *and *F. vesca *were retrieved from TAIR http://www.arabidopsis.org, PlantGDB http://www.plantgdb.org/VvGDB/ and PFR Strawberry server http://www.strawberrygenome.org, respectively. For *C. cardunculus*, the K-mer distribution was generated using the raw paired end sequence of "Creta 4" and its *de novo *assembled contigs. K-mers of length 10 nt were considered, and split according to the presence of 0, 1, 2 or more CpG. The "CpG rate" was estimated according as proposed by Karlin and Mrazek [32]:

$$p\left(CpG\right)=\frac{CpG}{p\left(C\right)p\left(G\right)}$$

where CpG represents the observed frequency of CpG dinucleotides and p(C) and p(G) the respective frequencies of each single nucleotide.

### SNP discovery

MAQ software (v. 0.5.0) [42] was used to align the paired end reads in the "Creta 4" reference contig set. The alignment threshold was set to a maximum of three nucleotides mismatch between Illumina reads and the reference. Gaps in the alignment of up to 2 nt allowed. Two levels of stringency were applied. In the first (CcRAD1), a comprehensive list of putative SNPs and 1-2 bp indels was populated with a minimum coverage of 6x as threshold prior to uploading to a Microsoft Access relational database; and for the second (CcRAD2), "fully informative" SNPs were defined when a minimum of 1-read allele calling was achieved for each of the three samples. In the latter set, heterozygous SNPs were assessed where the within sample allele frequency ranged from 0.25 to 0.75, together with a minimum coverage of 4x and allele calling for two reads. Sites were assigned as homozygous when the minor allele frequency fell below 0.10.

Candidate SNP markers were categorized as testcross in pair-wise comparisons of genotypes, whether a heterozygous imputation was present for one parent only (testcross) and a homozygous site was predicted for the other. Common intercross markers were defined for loci showing heterozygous states across all the three samples.

### CAPS assay design and application

A subset of heterozygous SNPs was selected from the "Altilis 41" sequence, and a search carried out for *Bam*HI, *Eco*RI, *Eco*RV, *Nde*I, *Xba*I, *Bcc*I, *Fok*I, *Xmn*I and *Dra*I (6 bp cutters), or *Taq*I and *Mse*I (4 bp cutters) recognition sites using SNP2CAPS script (v. 0.6) [43]. A predicted fragment size difference of at least 20 bp was imposed to allow detection on standard agarose gels. Locus-specific primers were designed from the BatchPrimer3 web interface [44], using default parameters but for product size (100-400 bp) and annealing sites (within a 50 bp window at either end of the RAD contig). The resulting assays were applied to a set of 94 F_1 _segregants from the cross "Romanesco C3" × "Altilis 41" [11]. PCRs were carried out in a 20 μl volume containing 12.5 ng genomic DNA, 1x GoTaq Buffer (Promega), 1.5 mM MgCl_2_, 0.2 mM dNTPs, 1 U GoTaq (Promega) and 0.5 μM of each primer. The cycling regime was 95°C/5 min, followed by 35 cycles of 95°C/30 s, 55°C/30 s, 72°C/45 s and a final incubation of 72°C/5 min. Amplification was checked by electrophoresis through a 1.5% agarose gel and quantified using a Beckman Coulter spectrophotometer. Restriction reactions (20 μl) comprised 800 ng amplified DNA, 0.3 U restriction enzyme (New England Biolabs), reaction buffer and BSA according to the manufacturers' specifications, incubated for 4 h at 37°C (except for *Taq*I, where the incubation temperature was 65°C), after which the reactions were heat inactivated (80°C/10 min). The resulting products were electrophoresed through 2% agarose gels.

### Linkage analysis

The CAPS derived genotypic data were incorporated into a pre-existing data set of 273 molecular loci, mainly AFLP and EST-SSRs, already used to generate the cultivated cardoon genetic map [11,14,15] including five SNP from genes underlying caffeoylquinic acids synthesis reported by Comino et al. [45] and Menin et al. [46]; all maps data are available on request by the authors. Goodness-of-fit between observed and expected segregation ratios was tested by χ^2 ^and only markers fitting or deviating only marginally from expectation (χ^2^_α = 1 _< χ^2 ^≤ χ^2^_α = 0.01_) were included for mapping. Linkage groups (LGs) were established by JoinMap v4.0 software [47], on the basis of a LOD threshold of 6.0, using as parameter settings Rec = 0.40, LOD = 1.0, Jump = 5. Map distances were converted to centiMorgans (cM) using the Kosambi mapping function. LGs were drawn and aligned using MapChart v2.1 [48].

## Authors' contributions

SK and SL planned and supervised the experimental work; DS carried out the bioinformatic analysis; DS and MT performed the genotyping of the progenies; EP carried out linkage analyses and map construction; AA and EP and SL supervised the drafting of the manuscript. All authors read and approved the final manuscript.

## Supplementary Material

Additional file 1 **The data provided represent the assembled RAD contigs in fasta format**. RAD contigs. Click here for file

Additional file 2 **The data provided represent the list of the RAD contigs which were annotated with the Blast2Go pipeline**. Contigs annotation. Click here for file

Additional file 3 **The data provided represent the list of the RAD contigs which were mapped in the KEGG's pathway**. KEGG's pathways mapping. Click here for file

Additional file 4 **Distributions of K-mers using random dinucleotides**. distributions of K-mers. Click here for file

Additional file 5 **Comprehensive list of SNPs and 1-2 bp indels**. CcRAD1 SNP list. Click here for file

Additional file 6 **list of the "fully informative" SNPs and test cross markers**. CcRAD2 SNP list. Click here for file
