# Supplementary material for: RAD tag sequencing as a source of SNP markers in Cynara cardunculus L
Source: BMC Genomics. 2012 Jan 3;13:3. doi: 10.1186/1471-2164-13-3 (PMC3269995; doi:10.1186/1471-2164-13-3)

Cynara cardunculus pre-assembly

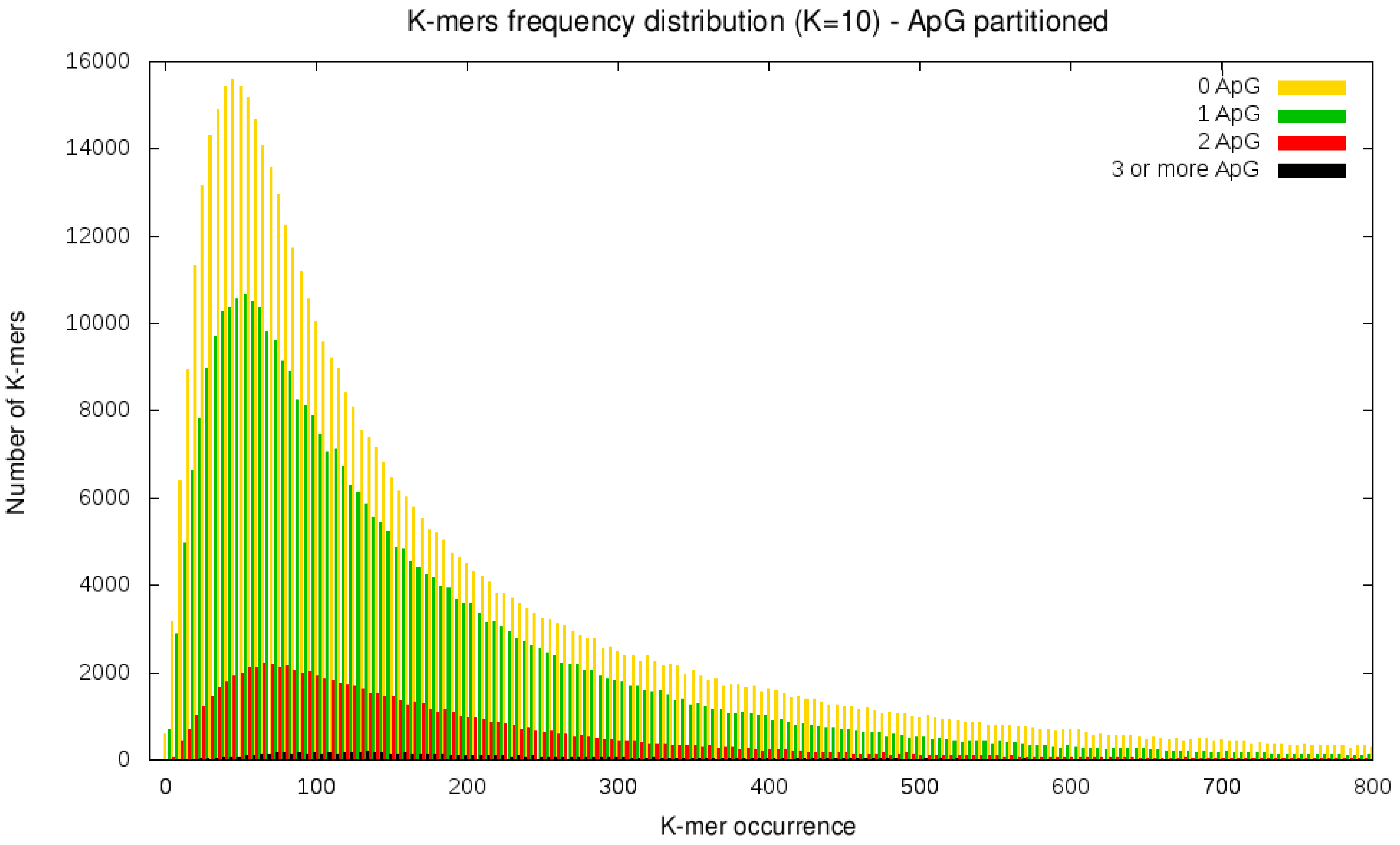

Arabidopsis thaliana genome assembly

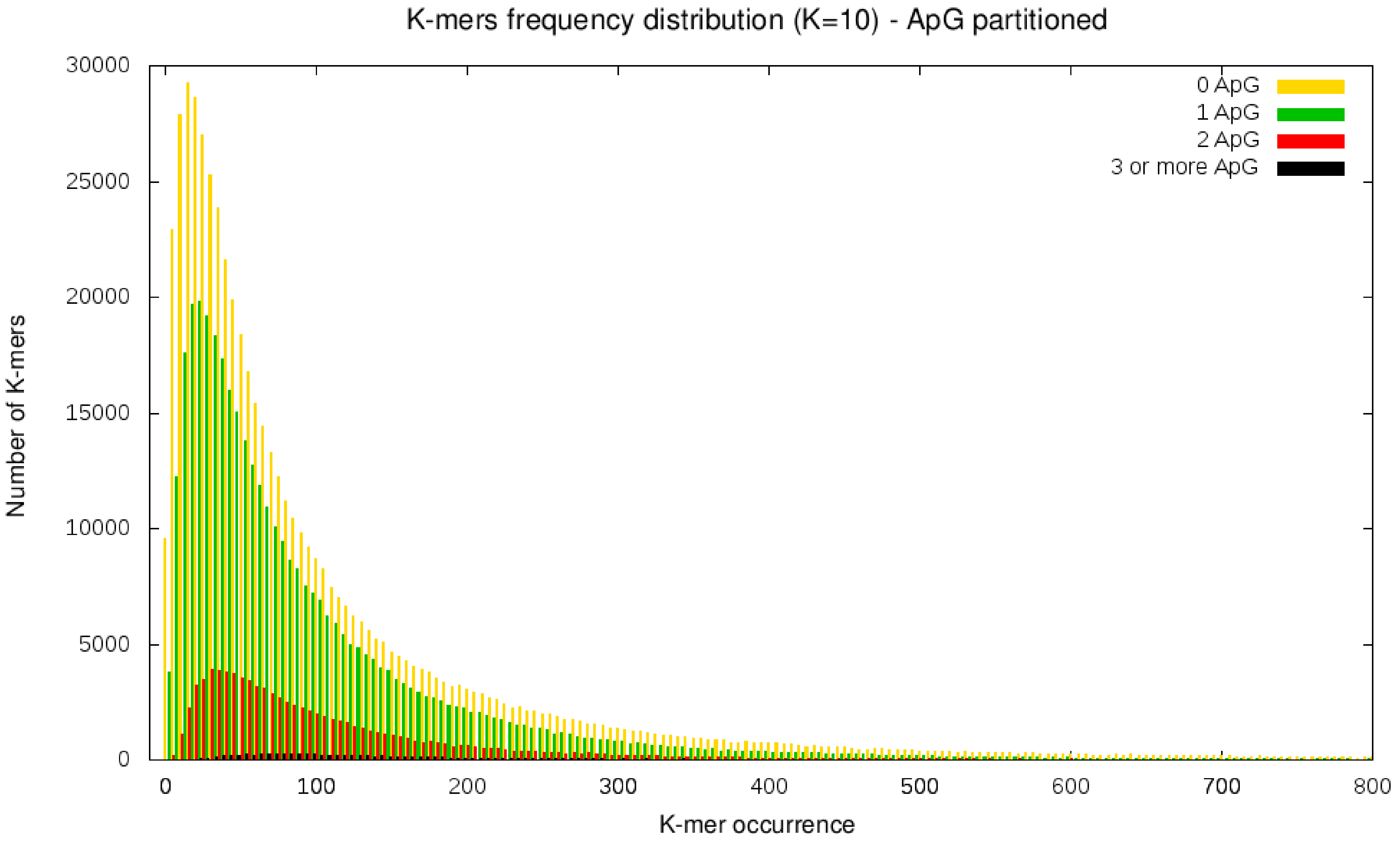

Arabidopsis thaliana genome assembly

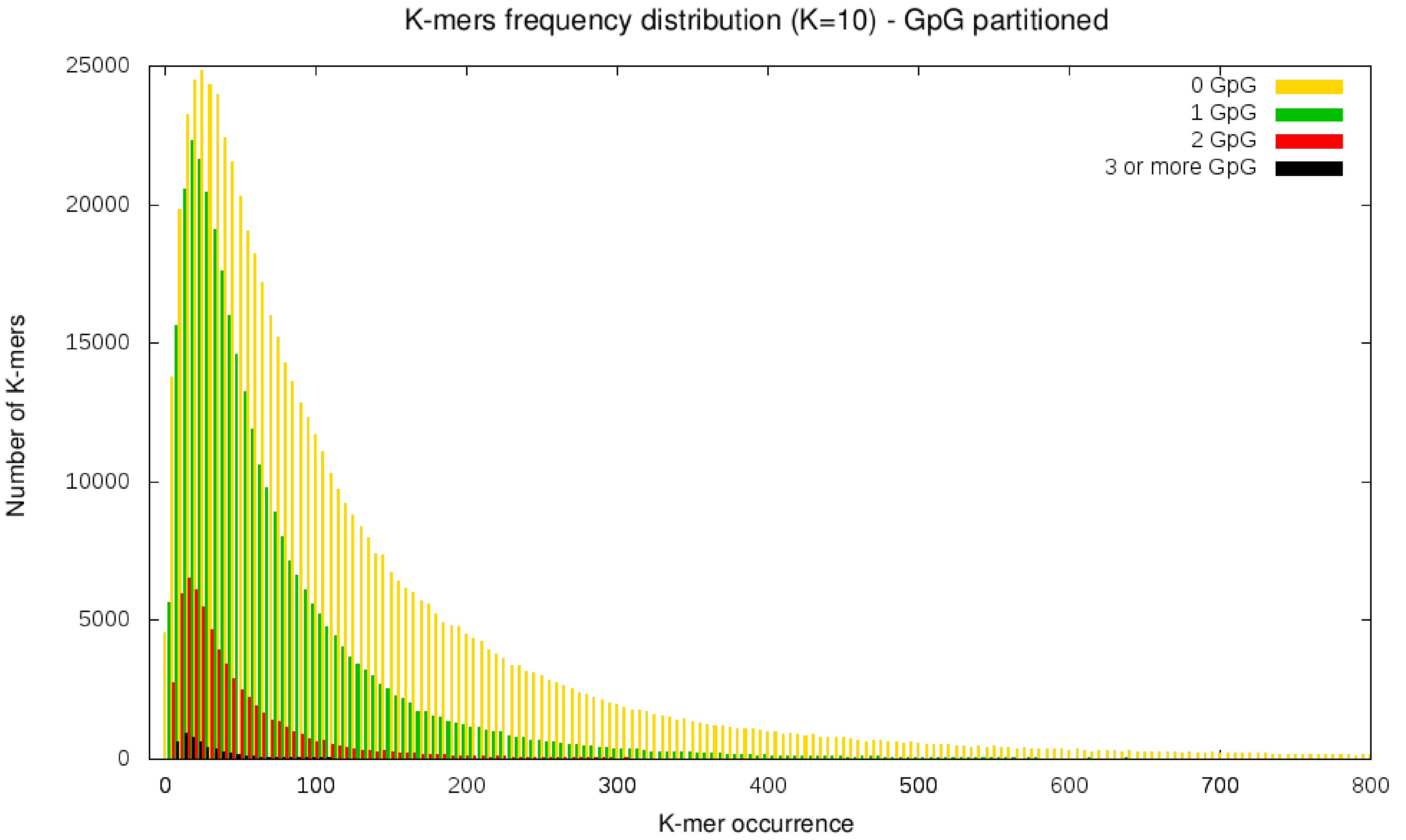

Supplement: Additional file 4 — Distributions of K-mers using random dinucleotides. distributions of K-mers. [file 1471-2164-13-3-S4.PDF]
